# Fulminant *Clostridium* *difficile* Enteritis after Proctocolectomy and Ileal Pouch-Anal Anastamosis

**DOI:** 10.1155/2008/985658

**Published:** 2009-02-01

**Authors:** Elena Boland, Jon S. Thompson

**Affiliations:** Department of Surgery, University of Nebraska Medical Center, 983280 Nebraska Medical Center Omaha, NE 68198-3280, USA

## Abstract

*Clostridium difficile* (*C. difficile*) infection of the small bowel is very rare. The disease course is more severe than that of *C. difficile* colitis, and the mortality is high. We present a case of *C. difficile* enteritis in a patient with ileal pouch-anal anastamosis (IPAA), and review previous case reports in order to better characterize this unusual condition.

## 1. INTRODUCTION

*C. difficile* infection is associated with antibiotic-induced pseudomembranous colitis. This infection is usually thought to be restricted to the colon. Isolated small bowel *C. difficile* enteritis is rare and can manifest in the absence of a colon.

*C. difficile* has been shown to colonize small bowel mucosa in about 3% of the population, which then serves as a reservoir for infection [1]. Most carriers are asymptomatic. Altered intestinal anatomy and antibiotic use have been implicated in triggering symptomatic infection. Fecal flora in the small bowel of patients who had undergone a colectomy is altered to resemble that of the colon. Morphological changes (colonic-type metaplasia with partial villous atrophy) which occur in the mucosa of an ileal pouch secondary to altered fecal flow may predispose to infection [2]. These factors may increase small bowel colonization by *C. difficile*. Alteration of fecal flora by antibiotic can trigger symptomatic infection.

The clinical presentation of *C. difficile* colitis is typically mild, occasionally progressing to fulminant colitis. The disease course is more fulminant when small bowel is affected, with reported mortality ranging from 60–83% [3]. We report a case of fulminant *C. difficile* enteritis in a patient with ileal pouch-anal anastamosis (IPAA), and review previous reports of this unusual condition.

## 2. REPORT OF A CASE

**Figure 1:**
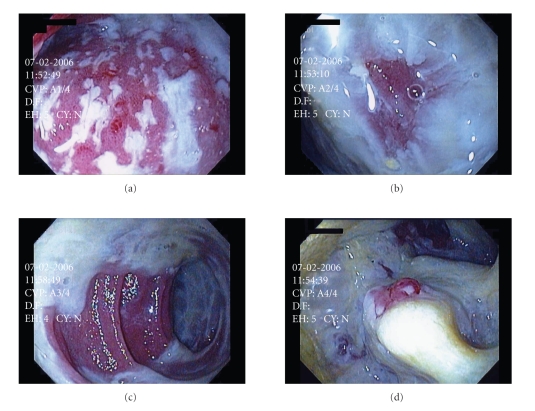
Flexible endoscopy of pelvic pouch demonstrating copious amounts of mucus with adherent pseudomembranes throughout the pouch and distal small bowel, consistent with *C. difficile* infection.

A 42-year-old man underwent proctocolectomy with IPAA and ileostomy for medically refractory ulcerative colitis (UC). The patient returned for ileostomy takedown six months later. His hospital course was complicated by a urinary tract infection, which was treated with ciprofloxacin. The patient was discharged tolerating a regular diet with good bowel function. The patient returned 10 days later complaining of a three-day history of nausea, diarrhea, and abdominal pain. Patient was febrile (38.3°C), tachycardic (138), with elevated white blood cell count (17.000), creatinine (2.5), and platelet count (1450). CT scan of the abdomen showed dilated small bowel with fluid and air to the ileoanal anastomosis. Blood, urine, and stool cultures were sent, and empiric intravenous piperacillin/ tazobactam and vancomycin were started. However, the patient became progressively more septic and required vasopressors for blood pressure support. Unexpectedly, *C. difficile* enzyme immunoassay (EIA) came back positive for toxins A and B, and the patient started on oral vancomycin and metronidazole. Flexible endoscopy was performed and revealed copious amounts of mucus with adherent pseudomembranes throughout the pouch and distal small bowel (Figure 1). Over the next few days, the patient remained in critical condition, but then slowly stabilized. Vasopressors were weaned; WBC and creatinine came down to normal limits. Within 7 days of admission, patient was restarted on a diet and was ultimately discharged after a 12-day hospitalization. At one year follow up, the patient still occasionally has frequent bowel movements, but his stool cultures have remained negative for *C. difficile* toxins.

## 3. DISCUSSION

PubMed literature search for *C. difficile* enteritis was performed and revealed 26 cases from 1980–2008 (Table 1) [3–22]. There was significant age variability, with a range of 18 to 83 years of age (mean 50.3). Sixteen of the 26 patients had inflammatory bowel disease (IBD), thirteen patients had ulcerative colitis, and three had Crohn's disease. Ten patients had total colectomies and six underwent IPAA. All but three patients had altered intestinal anatomy. Twenty four patients had recent hospitalization and/or operation as well as recent antibiotic use. Thirteen patients were septic and required ICU admission. In all 26 cases, the stool assays were positive for *C. difficile* toxin. Diagnosis of small bowel involvement was made based on biopsy, pathology, or autopsy results. Only seven patients were evaluated endoscopically. Four underwent flexible sigmoidoscopy, and three of those had pseudomembranes. Of the patients with IPAA, only two were examined endoscopically and no pseudomembranes were visualized. One patient had an esophagogastroduodenoscopy (EGD) which demonstrated pseudomembranes in the duodenum. Treatment in all but two patients included metronidazole or vancomycin, or a combination of both. Two patients were resistant to metronidazole. Fourteen of the 26 underwent operative intervention. Mortality rate was 35%.

Our patient was similar to the previously reported cases of *C. difficile* enteritis in that he had a history of IBD, recent surgery, and antibiotic use. He required ICU admission secondary to sepsis, but he did not require operative intervention. Unlike any of the previously reported cases, our patient's pouch endoscopy revealed pseudomembranes, facilitating timely intervention and his ultimate recovery.

*C. difficile* enteritis appears to have a fulminant course, with high risk of sepsis, need for operation, and mortality. It is unclear why the disease course is more severe than in colitis. Increased small bowel permeability is one potential explanation. Delay in diagnosis and treatment may play a role as well.

The clinical presentation can be similar for both enteritis and colitis. Symptoms include diarrhea, dehydration, and increased ileostomy output. Unlike colitis, enteritis more commonly presents with systemic manifestations such as fever, hypotension, leukocytosis and thrombocytosis [3], and occasionally with peritonitis or bowel perforation [7, 17].

*C. difficile* enteritis may be difficult to differentiate from other inflammatory processes, and requires high degree of suspicion to make the diagnosis. *C. difficile* has also been implicated as a cause of chronic pouchitis in patients with IPAA [16], and should be suspected in this setting. Given the higher risk that IBD patients may have for developing *C. difficile* enteritis, it is important to be able to differentiate it from an exacerbation of IBD. Diagnosis is made by identifying *C. difficile* toxin A or B in the stool. Similarly, endoscopy should be utilized in patients with suspected small bowel involvement even with history of prior colectomy. This may facilitate differentiation between Crohn's enteritis, pouchitis, and *C. difficile* enteritis.

As with our patient, most cases will respond to treatment with metronidazole or vancomycin. However, more virulent and resistant strains have been reported [23]. Some patients will need emergent surgical resection of any perforated or gangrenous bowel if they fail to respond to medical treatment.

*C. difficile* enteritis is emerging with increased frequency and can have devastating results. Patients with IBD and prior colectomy are at increased risk. Prompt identification of the organism via stool culture and endoscopy may result in more favorable outcomes.

## Figures and Tables

**Table 1:** Previously reported cases of small bowel *C. difficile*.

	Author	Age	IBD	Intestinal operation	Recent hospitalization/operation	Recent Abx	ICU/Sepsis	OR	Endoscopy	Treatment	Death	Notes
1	LaMont and Trnka [4] 1980	23	Crohn's	Partial colectomy	No	No	No	No	EGD—pseudomembranes in duodenum	Vancomycin	No
2	Shortland et al. [5] 1983	70	No	Ileal conduit	Yes	Yes	—	No	Sigmoidoscopy—pseudomembranes	Vancomycin	Yes	
3	Testore et al. [6] 1984	69	No	APR	Yes	Yes	Yes	No	—	—	Yes	
4	Miller et al. [7] 1989	18	No	—	Yes	Yes	Yes	Yes	Flexible sigmoidoscopy—inflammation; no pseudomembranes	Streptomycin	No	2 jejunal perforations
5	Kuntz et al. [8] 1993	53	UC	TAC	Yes	Yes	Yes	Yes	—	Vancomycin, flagyl	Yes	Intramural gas on CT
6	Tsutaoka et al. [9] 1994	66	No	Rt hemicolectomy + APR	Yes	Yes	Yes	Yes	—	Vancomycin, flagyl	Yes	
7	Yee et al. [10] 1996	71	No	TAC	Yes	Yes	Yes	Yes	—	Flagyl	Yes	
8	Kralovich et al. [11] 1997	65	No	Jejunal-ileal bypass	Yes	Yes	Yes	Yes	Flexible sigmoidoscopy—pseudomembranes	Vancomycin, flagyl	Yes	
9	Vesoulis et al. [12] 2000	56	Crohn's	TPC	Yes	Yes	Yes	Yes	—	Flagyl	No	
10	Freiler et al. [13] 2001	26	UC	TAC	Yes	Yes	No	No	—	Flagyl	No	
11	Jacobs et al. [14] 2001	83	—	—	Yes	Yes	—	Yes	—	—	No	
12	Tjandra et al. [15] 2001	60	No	Sigmoid colectomy	Yes	Yes	Yes	Yes	Flexible sigmoidoscopy—pseudomembranes	Vancomycin, flagyl	Yes	
13	Mann et al. [16] 2003	35	UC	IPAA	No	No	No	No	Flexible endoscopy—inflammed, ulcerated mucosa	Vancomycin; resistant to flagyl	No	Chronic pouchitis
14	Hayetian et al. [17] 2006	80	No	LAR	Yes	Yes	Yes	Yes	—	Flagyl	Yes	Ileal perforation
15	Hayetian et al. [17] 2006	83	No	None	Yes	Yes	Yes	Yes	—	Vancomycin, flagyl	No	Ileal perforation
16	Kim et al. [18] 2007	65	Crohn's	TPC	Yes	Yes	Yes	No	—	Flagyl	Yes	
17	Lundeen et al. [3] (6 patients) 2007	Mean 35.3	UC (6 patients)	3 IPAA 3 TAC	6/6	6/6	1/6	1/6	—	Vancomycin, flagyl	No	
18	Wood et al. [19] 2008	48	UC	IPAA	Yes	Yes	Yes	Yes	Flexible endoscopy—normal pouch	Flagyl	No	
19	Follmar et al. [20] 2008	49	UC	IPAA	Yes	Yes	No	Yes	—	Vancomycin, resistant to flagyl	No	Or–mesh removal
20	Fleming et al. [21] 2008	54	UC	TAC	Yes	Yes	?	No	—	Flagyl, vancomycin, rifampin	No	
21	Yafi et al. [22] 2008	21	UC	TAC	Yes	Yes	?	Yes	—	Vancomycin	No	Pelvic abscess

	Total	50.3	16/26	23/26	24/26	24/26	13/26	14/26	7/26	21/26	9/26	

## NOMENCLATURE

APR: Abdominoperineal resection
TAC: Total abdominal colectomy
TPC: Total proctocolectomy
IPAA: Ileal pouch-anal anastamosis.
